# Experiences of Health Facility Childbirth in Sub-Saharan Africa: A Systematic Review of Qualitative Evidence

**DOI:** 10.1007/s10995-022-03383-9

**Published:** 2022-02-26

**Authors:** Uchenna Gwacham-Anisiobi, Aduragbemi Banke-Thomas

**Affiliations:** 1 Department of Population Health, University of Oxford, Oxford, UK; 2 Department of Public Health, University of Liverpool, Liverpool, UK; 3 Department of Health Policy, London School of Economics and Political Science, London, UK; 4 School of Human Sciences, University of Greenwich, Greenwich, London, UK

**Keywords:** Maternal health, Quality of care, Experience of care, Sub-Saharan Africa, Facility childbirth, Institutional births

## Abstract

**Introduction:**

Access to skilled birth attendance has been prioritised as an intervention to minimise burden of maternal deaths in sub-Saharan Africa (SSA). However, poor experience of care (EoC) is impeding progress. We conducted a systematic review to holistically explore EoC patterns of facility-based childbirth in SSA.

**Methods:**

PubMed, Embase and Scopus databases were searched to identify SSA EoC studies conducted between January 2000 and December 2019. Studies meeting our pre-defined inclusion criteria were quality assessed and relevant data extracted. We utilised the EoC quality standards (defined by the World Health Organization) to summarise and analyse findings while highlighting patterns.

**Results:**

Twenty-two studies of varying quality from 11 SSA countries were included for review. Overall, at least one study from all included countries reported negative EoC in one or more domains of the WHO framework. Across SSA, ‘respect and preservation of dignity’ was the most reported domain of EoC. While most women deemed the pervasive disrespect as unacceptable, studies in West Africa suggest a “normalisation” of disrespect, if the intent is to save their lives. Women often experienced sub-optimal communication and emotional support with providers in public facilities compared to non-public ones in the region. These experiences had an influence on future institutional deliveries.

**Discussion:**

Sub-optimal EoC is widespread in SSA, more so in public facilities. As SSA heath systems explore approaches make progress towards the Sustainable Development Goal 3, emphasis needs to be placed on ensuring women in the region have access to both high-quality provision and experience of care.

**Supplementary Information:**

The online version contains supplementary material available at 10.1007/s10995-022-03383-9.

## Significance Statement

Several studies have documented experiences of care during health facility childbirth in sub-Saharan Africa. However, only two previously published reviews have synthesised the available evidence, and these reviews only explored one of the three experience of care domains as stipulated in the World Health Organization’s Quality of Care framework. Our systematic review provides the first holistic assessment of the experience of care during health facility childbirth in the region, along the three domains of effective communication, respect and preservation of dignity, and emotional support. Findings provide much needed evidence synthesis that will be critical for improving care experiences of women.

## Introduction

Globally, an estimated 295,000 maternal deaths occur annually, with about 66% of these deaths occurring in sub-Saharan Africa (SSA) (WHO et al., 2019). Though access to quality maternity care reduces over a third of these deaths (WHO, 2004), the rate of skilled birth attendance and facility-based deliveries in SSA still pales at 59% and 22%, respectively (Doctor et al., 2018). Evidence suggests that negative care experiences during facility-based births may deter future use by affected women and those within their social circles (Afulani et al., 2017; Ishola et al., 2017).

**Fig. 1:**
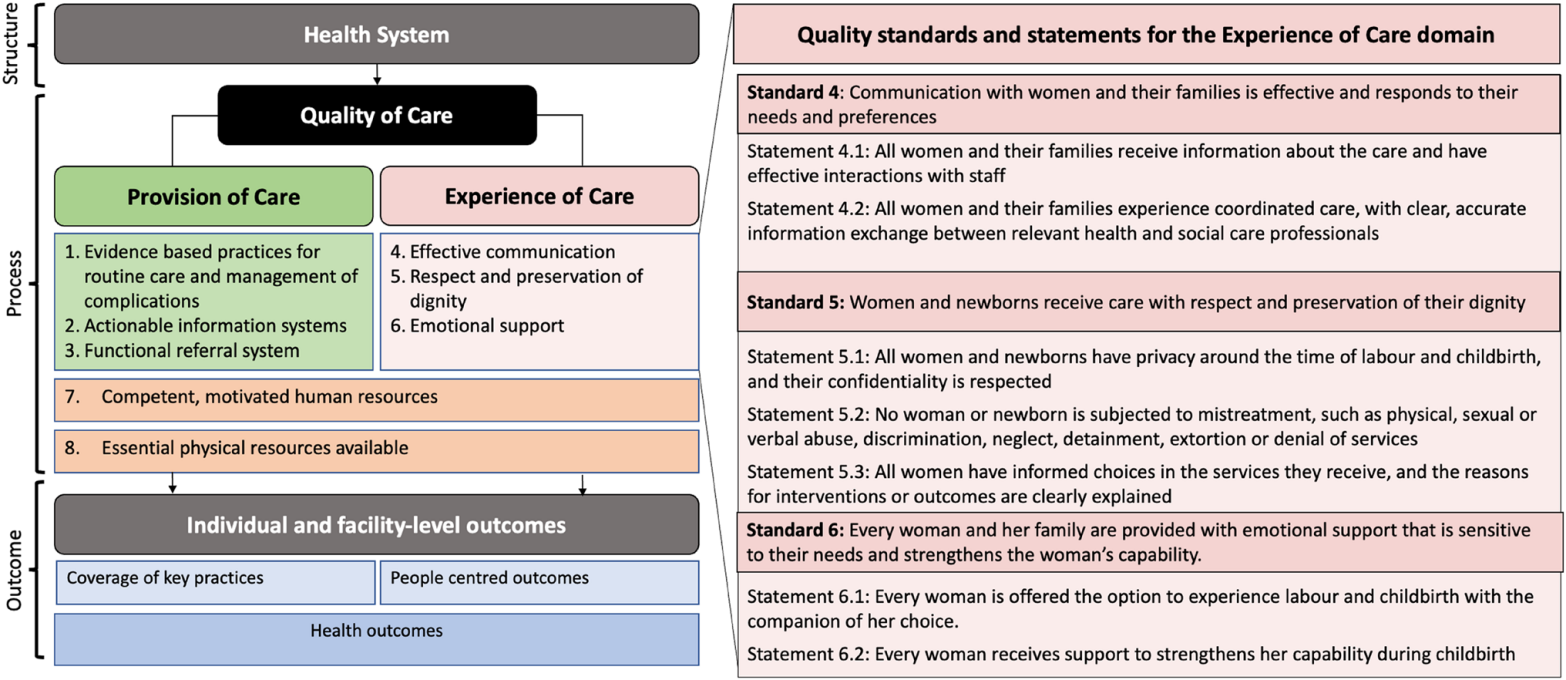
World Health Organization’s quality of care framework for maternal and newborn health with quality standards and statements for experience of care

The World Health Organization (WHO) characterises care experience within its Quality of Care (QoC) framework for maternal and newborn health (WHO, 2016). The broad vision of this framework is to equally prioritise provision and experience of care (EoC), increase facility-based deliveries, and attain and sustain positive outcomes (Tunçalp et al., 2015). The EoC dimension of this framework has three domains (effective communication, respect and dignity, and emotional support) each with defined standards of care and a few specific quality statements (Fig. 1) (WHO, 2016).

Two systematic reviews exploring EoC in SSA have been published to date, with one being specific to the region (Bradley et al., 2016) and the other part of a global study (Bohren et al., 2015). Both reviews focused on disrespect/maltreatment of women during facility childbirth which relates to one of the three domains. In addition to serving as an update to these earlier single domain-focused reviews, our systematic review expands scope to critically assess and synthesize peer-reviewed studies that explore care experience in SSA across all three EoC domains. Our review also sets out to explore observable patterns of EoC during childbirth in SSA.

## Methods

A qualitative systematic review was conducted in accordance with the Preferred Reporting Items for Systematic Reviews and Meta-Analyses guidelines (Moher et al., 2009) (Supplementary file 1).

Using the sample-phenomenon of interest-design-evaluation-research type search strategy, which is preferred for qualitative article retrieval (Cooke et al., 2012), search terms and synonyms were generated (Supplementary file 1). PubMed, Embase and Scopus databases were searched to identify articles published between January 2000 and December 2019. Search terms capturing sample (women), phenomenon of interest (facility-based childbirth), design (EoC), evaluation (experience) research type (qualitative or mixed) were applied, using Boolean operators “OR” within each category and “AND” between categories of search terms. Duplicates were identified and removed. Reference list checking was done to determine other relevant materials which may have been missed in the initial search. Title and abstract screening were conducted for article relevance.

We included full-text articles meeting the inclusion criteria of being peer-reviewed qualitative or mixed-methods studies that explored at least one of the three EoC domains in SSA. Included studies also needed to be conducted from the perspectives of women or their relatives. Studies that captured EoC from the perspective of skilled health personnel (SHP) including nurses/midwives and doctors (WHO et al., 2018), commentaries, conference proceedings, editorials and quantitative studies were excluded. In instances of disagreement regarding the inclusion of specific full-text articles, this was resolved through negotiations.

Both authors independently appraised quality of included studies using the 10-criterion Critical Appraisal Skills Programme checklist (CASP, 2018). A score of ‘1’ was given for each satisfied criterion and ‘0’ if the criterion was not met. Studies achieving 7–10 points were rated as high quality. Those achieving 5–6 points were moderate quality while < 5 points were rated as low quality.

The authors read all included studies in detail to extract pertinent data for the review using a pre-designed extraction sheet. Data extracted included: names of author(s), country, recruitment settings (in-facility/community), scale of study (multinational/national/sub-national/local), facility ownership (public/private/mission/military), sample size, participant selection and findings presented for the EoC domain explored. We summarised the descriptive characteristics of the included studies, using tables and charts. Both authors independently assessed the findings for each of the quality statements in the EoC domain and subsequently deliberated on their subjective assessment of the rating for each quality statement, as defined by the WHO (WHO, 2016). The ratings were done on a five-point Likert scale ranging from ‘very poor’ to ‘very good’. Any quality statement not reported in each paper was rated ‘NR’. We then used a five-colour coding system to represent the assessed scale. A framework synthesis approach (Barnett-Page & Thomas, 2009), based on the WHO QoC framework (WHO, 2016), was used to synthesise findings. We described observed patterns from the available evidence, taking a largely deductive approach.

## Results

**Fig. 2:**
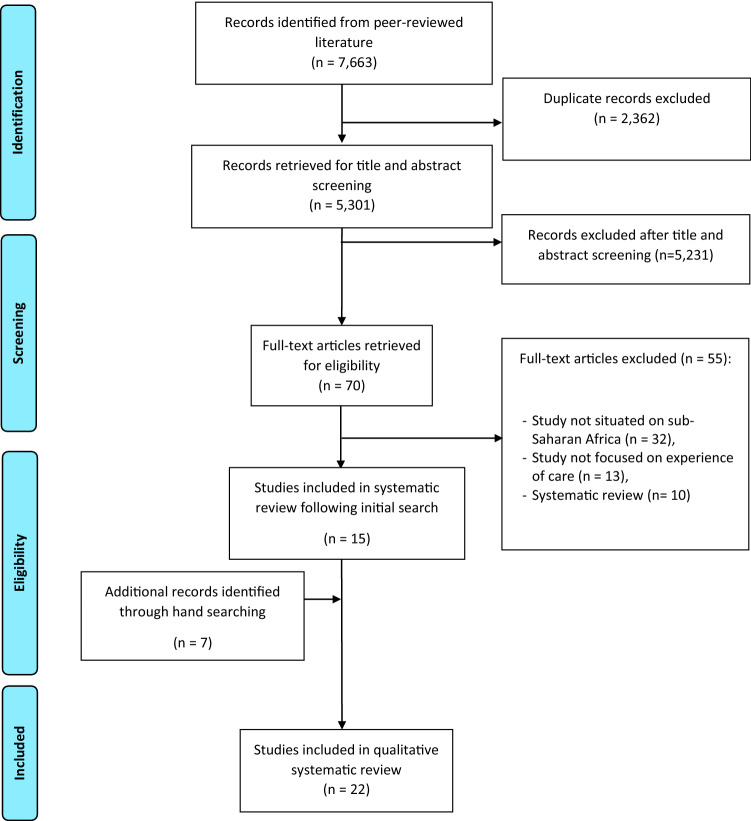
PRISMA flow diagram of the literature review process

The electronic search yielded 7663 results. Following removal of duplicates, 5,301 results remained, from which 70 records were retained, after title and abstract screening. Following full-text assessment, 22 (21 qualitative quality Adinew & Assefa, 2017; Afulani et al., 2017; Balde et al., 2017; Bohren et al., 2016, 2017; D’Ambruoso et al., 2005; Dzomeku et al., 2017; Kumbani et al., 2012; Madula et al., 2018; Maputle & Nolte, 2008; Maya et al., 2018; McMahon et al., 2014; Mensah et al., 2014; Mukamurigo et al., 2017; Muntenda et al., 2017; Namujju et al., 2018; Ojelade et al., 2017; Okonofua et al., 2017a, b; Okwako & Symon, 2014; Orpin et al., 2018; Shimpuku et al., 2013) and one mixed (Nwosu et al., 2012) studies met the inclusion criteria (Fig. 2).

Of the 22 studies, fifteen were adjudged as high-quality (Adinew & Assefa, 2017; Afulani et al., 2017; Balde et al., 2017; Bohren et al., 2016, 2017; Dzomeku et al., 2017; Kumbani et al., 2012; Maya et al., 2018; McMahon et al., 2014; Mukamurigo et al., 2017; Muntenda et al., 2017; Namujju et al., 2018; Ojelade et al., 2017; Okwako & Symon, 2014; Orpin et al., 2018), six as moderate quality (D’Ambruoso et al., 2005; Madula et al., 2018; Maputle & Nolte, 2008; Mensah et al., 2014; Okonofua, et al., 2017a, b; Shimpuku et al., 2013) and one low-quality (Nwosu et al., 2012) (Table 1 and Supplementary file 1).

**Table 1:** Characteristics and quality assessment of included studies

S/N	Authors	Date	Country	Participant selection	Sample size	Type of health facility women used	Place of recruitment	Scale of study	Study design	CASP score (n/10)
1	Adinew, and Assefa	2017	Ethiopia	Purposive	16 KIIs and 8 FGDs	Unclear	Communities	Subnational	Qualitative	9
2	Afulani, Kurmbi and Lyndon	2017	Kenya	Purposive	8 FGDs	86% public facilities, 9% private/mission, 5% others	Communities	Subnational	Qualitative	8
3	Balde et al	2017	Guinea	Purposive	40 IDIs and 8 FGDs	Public	Communities	Local	Qualitative	9
4	Bohren et al	2016	Nigeria	Stratified purposive (quota sampling)	41 IDIs and 4 FGDs	Unclear	Communities	Local	Qualitative	10
5	Bohren et al	2017	Nigeria and Uganda	Purposive—maximum variation	132 IDIs and 21 FGDs	Mostly public	Communities	Multinational	Qualitative	8
6	D'ambruso, Abbey & Hussein	2005	Ghana	Opportunistic (participants of another study)	21 IDIs and 2 FGDs	Mostly public	Health facilities	Subnational	Qualitative	6
7	Dzomeku, van Wyk, and Lori	2017	Ghana	Purposive	56 IDIs	Public	Health facilities	Local	Qualitative	8
8	Kumbani et al	2012	Malawi	purposive	14 IDIs	Public	Health facility	Local	Qualitative	9
9	Madula et al	2018	Malawi	Systematic random sampling	6 hospitals in the three regions of Malawi	50% public 50% private	Health facilities	National	Qualitative	6
10	Maputle and Nolte	2008	South Africa	convenience	24 IDIs	Public	Health facility	Local	Qualitative	5
11	Maya et al	2018	Ghana	Purposive	41 IDIs, 10 FGDS	Mostly public	Communities	Local	Qualitative	9
12	McMahon et al	2014	Tanzania	Purposive	49 IDIs	unclear	Communities	Subnational	Qualitative	9
13	Mensah, Mogale and Ritcher	2014	Ghana	Purposive	9 IDIs	Military	Health facility	Local	Qualitative	6
14	Mukamurigo et al	2017	Rwanda	Second level following a cross sectional survey	17 IDIs	Public	Communities	Local	Qualitative	8
15	Muntenda, Nuuyoma and Stern	2017	Namibia	Purposive	3 FGDs	Public	Health facility	Local	Qualitative	7
16	Namujju et al	2018	Uganda	Purposive	12 IDIs and 2 FGDs	Public	Health facility	Local	Qualitative	8
17	Nwosu et al	2012	Nigeria	Unclear	8 FGDs	72.8% private facilities 15.6% public	Communities	Local	Mixed methods	**4**
18	Ojelade et al	2017	Nigeria	Stratified purposive (quota sampling)	42 IDIs and 10 FGDs	unclear	Communities	Local	Qualitative	9
19	Okonofua et al	2017	Nigeria	Unclear	40 FGDs	public	Health facilities	National	Qualitative	6
20	Okwako and Symon	2014	Kenya	Purposive	7 IDIs	Public	Health facility	Local	Qualitative	7
21	Orpin et al	2018	Nigeria	purposive	5 FGDs	Public	Health facilities	Local	Qualitative	8
22	**S**himpuku et al	2013	Tanzania	Maximum variation	25 IDIs	Mission	Health facility	Local	Qualitative	6

### Characteristics of Included Studies

**Fig. 3:**
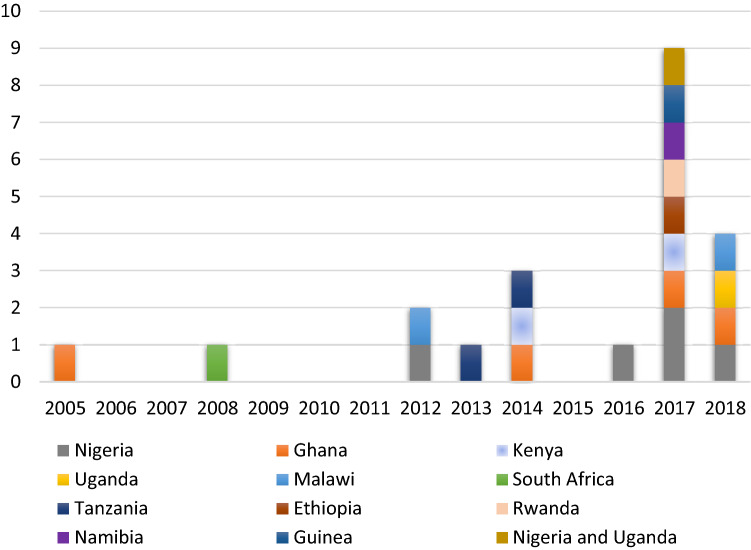
Distribution of included studies by year of publication

**Fig. 4:**
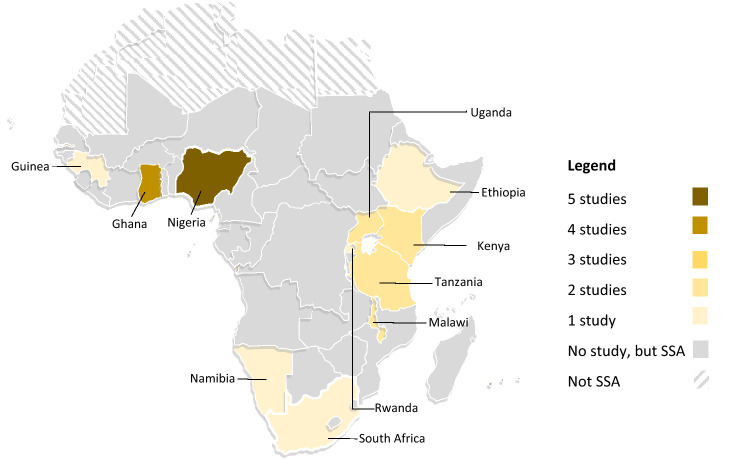
Map of Africa indicating the countries with published literature

An average of one study was published in each year, with the peak publication in 2017 having nine studies (Table 1 and Fig. 3). The included studies were conducted in 11 SSA countries located in three of the four SSA sub-regions, excluding Central Africa. Ten studies originated from six East African countries (Kenya Afulani et al., 2017; Okwako & Symon, 2014), Uganda (Bohren et al., 2017; Namujju et al., 2018), Malawi (Kumbani et al., 2012; Madula et al., 2018), Tanzania (McMahon et al., 2014; Shimpuku et al., 2013), Ethiopia (Adinew & Assefa, 2017), Rwanda (Mukamurigo et al., 2017)). Included studies from West Africa were conducted in Nigeria (Bohren et al., 2016, 2017; Nwosu et al., 2012; Okonofua et al., 2017a, b; Orpin et al., 2018), Ghana (D’Ambruoso et al., 2005; Dzomeku et al., 2017; Maya et al., 2018; Mensah et al., 2014), and Guinea (Balde et al., 2017). While studies from Southern Africa were from South Africa (Maputle & Nolte, 2008) and Namibia (Muntenda et al., 2017) (Fig. 4).

Twelve studies were facility-based and the other ten were conducted in the communities. Fourteen studies had women who all or mostly utilised public facilities. In the other studies, one had > 70% of women who used private facilities, another 50% of the women used public and private hospitals respectively, one had all women who used a mission hospital and another military hospital only. In four studies, the facility ownership was unclear (Table 2).

**Table 2:**
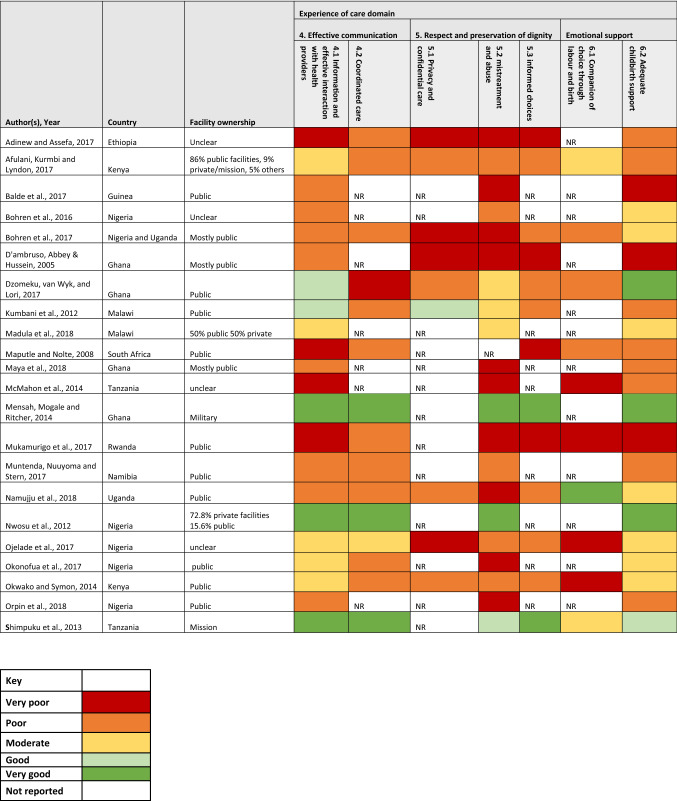
Summary table showing data from included studies

Additional characteristics regarding the included studies including objectives, context and timing of engaging women in the primary studies are presented in Supplementary file 1.

### Findings from Evidence Synthesis of the EoC Domains

Summary of the evidence synthesis for each EoC domain is presented in the ensuing paragraphs while supportive quotes extracted from the primary studies are in Supplementary file 1. Overall, the respect and preservation of dignity domain has been the most studied with all included studies reporting on quality statement 5.2 focused on mistreatment and abuse. Conversely, the emotional support domain has been the least studied (< 50% of included studies) (Table 2).

#### Effective Communication

Across studies, women expect good communication with SHP which should commence on their arrival at the facility. In studies assessed to have ‘very good’ communication, women reported that SHP introduced themselves, spoke to them nicely, were responsive to their questions, and provided periodic updates on their progress (Dzomeku et al., 2017; Kumbani et al., 2012; Mensah et al., 2014; Nwosu et al., 2012; Shimpuku et al., 2013). However, 17 studies showed sub-optimal experience with communication (Table 2) with some women in these studies describing communication with SHP as “incomplete”, “unclear”, “tardy”, and “sometimes inconsistent across different shifts” (Adinew & Assefa, 2017; Afulani et al., 2017; D’Ambruoso et al., 2005; Kumbani et al., 2012; Maputle & Nolte, 2008; Mukamurigo et al., 2017). With the exception of women who received care in a private facility in Malawi (Kumbani et al., 2012), women in studies conducted in non-public facilities reported better information sharing and care coordination compared to public ones (Mensah et al., 2014; Nwosu et al., 2012; Shimpuku et al., 2013). Women who reported well-coordinated care emphasised the seamless transition of care information across shifts and health facilities in cases of referral (Afulani et al., 2017) (Table 2).

A number of studies which recruited women who utilised public facilities reported that women felt left out in their care as they did not receive sufficient information to make choices for medical interventions suggested by SHP (Kumbani et al., 2012; Namujju et al., 2018; Ojelade et al., 2017). The lack of capacity to communicate with auditory-challenged women was highlighted in a Malawian study conducted in private facilities (Kumbani et al., 2012). In two studies, women felt that the personality of SHP on duty during their facility confinement influenced their experience (Afulani et al., 2017; Ojelade et al., 2017).

#### Respect and Preservation of Dignity

Studies that recruited women who mostly used non-public facilities generally reported an experience of respectful care, while mistreatment and abuse were widely reported in studies conducted in public facilities. Verbal abuse was the most reported form of abuse and was experienced or witnessed by respondents in at least one study from all eleven countries included in this review (Table 2).

The commonest form of verbal abuse reported was speaking to women in raised voices, with some women stating that SHP outrightly hurled abusive and demeaning words at them (Adinew & Assefa, 2017; D’Ambruoso et al., 2005; Kumbani et al., 2012; Madula et al., 2018; Maya et al., 2018; Muntenda et al., 2017; Namujju et al., 2018). Physical abuse such as slapping, pinching, or hitting on the thighs were also detailed in accounts of negative experiences from all three SSA regions (Bohren et al., 2017; McMahon et al., 2014; Muntenda et al., 2017; Namujju et al., 2018; Orpin et al., 2018). While one study in South Africa reported that women saw abuse as unacceptable (Maputle & Nolte, 2008), three West African studies reported that women have normalised and accepted abuse by SHP, and some saw it as an “*encouragement to push*” (Balde et al., 2017; Maya et al., 2018; Orpin et al., 2018). Studies conducted in East African countries reported that women in response to verbal abuse engage in verbal or physical altercations with SHP (Adinew & Assefa, 2017; Afulani et al., 2017; McMahon et al., 2014). To protect themselves, some women in two East African studies request to have their birth companions with them, so that they can double as *“security”* to protect them from SHP that may abuse them during childbirth (Mukamurigo et al., 2017; Shimpuku et al., 2013).

Privacy and confidentiality of care was also reported as important for women during facility births (Adinew & Assefa, 2017; Kumbani et al., 2012; Namujju et al., 2018). Women using public facilities across the three SSA regions reported a lack or minimal levels of privacy during confinement (Adinew & Assefa, 2017; Afulani et al., 2017; Bohren et al., 2017; Namujju et al., 2018). On the contrary, all participants in a Malawian study, which was conducted in one public facility reported good privacy attributed to the presence of curtains demarcating women’s beds (Kumbani et al., 2012). Regarding informed consent, only women who used mission and military hospitals felt they made adequately informed choices (Table 2).

#### Emotional Support

Women who delivered in non-public hospitals generally reported feeling emotionally supported during childbirth, contrary to the experience of those who delivered in public facilities (Table 2).

Only one study, conducted in Uganda, reported that women were allowed their chosen companion during labour (Namujju et al., 2018). The other studies reported unmet need for companionship with hospital policy (Muntenda et al., 2017) and inadequate privacy for women on admission (Adinew & Assefa, 2017; Afulani et al., 2017; Bohren et al., 2017; Namujju et al., 2018; Ojelade et al., 2017) reported as reasons why women were denied companionship. Women wanted their loved ones to provide spiritual support usually in the form of prayers to allay their fears during labour (Mensah et al., 2014; Namujju et al., 2018; Ojelade et al., 2017).

Women appreciated emotional support from SHP in the form of kind words, allowing them to hold their hands while pushing and adopting a *“motherly”* role as they guided them through labour especially in settings where family members were not allowed (Bohren et al., 2017; Ojelade et al., 2017). If women did not feel emotionally supported, they chose to endure it as long as it guarantees good outcomes (D’Ambruoso et al., 2005; Dzomeku et al., 2017).

## Discussion

As per evidence gathered from the review, the highest number of studies published in a year (nine) was in 2017, which coincides with the launch of the WHO QOC framework in the preceding year (WHO, 2016). The quality of included studies did not vary by country. However, studies which recruited participants from the communities, or those which had participants from greater than two facilities tended to yield greater insights about EoC. In all but two studies (Mensah et al., 2014; Nwosu et al., 2012), most women reported suboptimal care experiences in at least one EoC domain. Women in other world regions have also reported sub-optimal experiences of childbirth (Bohren et al., 2015).

For the effective communication domain, our review shows that of its two subdomains, information sharing has been studied more than care coordination in SSA. In terms of observable patterns for these subdomains, EoC was consistently rated ‘very good’ in included studies conducted with women who delivered in non-public facilities. Though two studies conducted amongst women who delivered in public facilities were rated ‘good’ for information sharing, the common thread across the SSA regions was poor communication and coordination in the public sector. The public facility-based studies also reported sub-optimal care coordination. This might relate to the sub-optimal referral networks seen in SSA public sector (Ameyaw et al., 2020; Banke-Thomas et al., 2021). Effective communication is a critical bedrock capable of influencing how women experience the other two domains of EoC (Afulani et al., 2017). There was no clear observable pattern for experience of communication by country, scale of study, site, or time of recruitment across included studies.

Regarding the respect and preservation of dignity domain, all forms of disrespect including physical and verbal abuse, non-consented clinical care, non-confidential care, discrimination, abandonment of care, and detention in facilities (Bowser & Hill, 2010), have been reported across SSA. Of all, verbal and physical abuse were the most widely reported forms of disrespect. This is probably because the mistreatment and abuse sub-domain is the most widely studied. Disaggregated by facility ownership, assessed sub-domains for respect and preservation of dignity were consistently rated ‘very good’ or ‘good’ for included studies conducted in non-public facilities. On the contrary, in public facilities, all but one study, in which women reported that they had curtains demarcating their ward spaces (Kumbani et al., 2012), reported sub-optimal experiences. In response, it appears there is a gradual normalisation of these practices of disrespectful care by some women, as reported in Nigeria, where they believe the SHP mean no harm and are only “*encouraging them to push*” (Orpin et al., 2018). In Nigeria, a SHP justified abuse saying *“by slapping their laps, the patient will know that truly you care for her”* (Bohren et al., 2016).

For the emotional support domain, its adequate childbirth support sub-domain has been more widely studied compared to the other sub-domain of having a companion of choice during labour and childbirth. Broadly, our review found a mixed picture when it comes to EoC for both sub-domains across public and non-public facilities. However, for studies conducted with women who used public referral facilities, though many women were unhappy with poor emotional support offered by SHP, some believed it was a coping mechanism for SHP who were overwhelmed with the large patient load. Despite their dissatisfaction, they still chose to access care at these centres due to the perceived aggregation of highly specialised SHP in these facilities (D’Ambruoso et al., 2005; Dzomeku et al., 2017; Okonofua, et al., 2017a, b; Wright et al., 2017). There was no other clear observable pattern by the assessed study characteristics.

### Implications for Policy, Practice, and Research

Our review highlights some key implications for policy and practice. For policy, governments need to take the lead in standardising care experience that women can expect in SSA countries. This might involve leveraging insights from private providers unique to individual country context. While some have called for training/retraining of SHP in SSA (Gwacham-Anisiobi & Banke-Thomas, 2020; Wright et al., 2017), the limited evidence on effectiveness of such trainings suggests that they do not lead to improvement in women experiences (Chang et al., 2018). We argue that working conditions and environments need to be optimised for SHPs to ensure that they feel best placed to provide the care that women deserve. In addition, guidelines that incorporate communication strategies for improving patient experience in SSA countries need to be designed, implemented, and enforced.

For practice, establishing responsive feedback systems through anonymous client exit surveys or engagement events with women, as was done by a public hospital in Lagos (LIMH, 2021), can incentivise good practice by SHP to ensure they focus on ensuring women have the best EoC. However, collection of feedback through surveys without discussion and operationalisation of findings will not make a difference (Wong et al., 2020). As evidenced in our review, women in SSA have continued to demand companionship of their loved ones through labour and childbirth. While the concerns raised in implementing such practices in LMICs have been documented including cost of redesigning wards (Kabakian-Khasholian & Portela, 2017), the value to women cannot be ignored. Effecting this practice may make SHP more accountable and reduce incidences of disrespectful care. Also, partnerships with the private sector to purchase and set up low-cost redesigns of labour wards (e.g., curtains), when hospitals are resource-constrained should also be explored. This also addresses privacy concern that women in SSA have expressed. For research, more studies are needed in SSA, as only 11 countries had primary studies published. In addition, more research is needed on EoC in private facilities and amongst women with special needs. Furthermore, we need to properly explore the mechanism of disrespectful care.

### Strengths and Limitations

A major strength of this study is that it is a comprehensive qualitative systematic review of all three EoC domains of the WHO QoC framework in SSA. Though we have presented a subjective assessment of the domains reported in the studies, independent assessment of each study and subsequent deliberation between authors made for more robust assessments. However, there are certain limitations to consider. First, we conducted our assessment based on peer-reviewed literature only, and not reports where private facilities may more likely publish results, thus reducing our capacity to assess EoC with private providers. Nonetheless, in countries where we reviewed studies from both public and non-public facilities, (for example, in Ghana (Maya et al., 2018; Mensah et al., 2014) and Nigeria (Nwosu et al., 2012), the pattern showing better EoC in non-public facilities remained consistent. Second, there were some inherent biases reported in the included studies. For example, courtesy bias (tendency for women not to fully state their unhappiness with services received in order not to offend the SHP) (Hameed et al., 2017) and ‘halo effect’ (wrong assessment of care received in the postpartum attributed to the joy of a successful birth in studies done soon after birth) (Bennett, 1985) may have influenced some reported findings. However, using the study characteristics data that we collected, we explored any potential effects of these biases and found no clear pattern.

## Conclusion

Sub-optimal EoC is widespread in SSA, more so in public facilities. For many women, it is the singular reason they choose to access future childbirth services with unskilled providers (Adinew & Assefa, 2017; Afulani et al., 2017; McMahon et al., 2014). As countries in SSA continue to rise to the challenge of the disproportionately high burden of maternal deaths in the region, increasing chances for skilled birth attendance in facilities will remain a critical strategy. However, women should not have to choose between having skilled/expertly care and respectful care. Both must be part of the care that women can expect and experience in SSA. If the focus remains achieving the Sustainable Development Goal 3, there is need for every woman who arrives at a health facility to feel welcomed, pampered and supported to go through what may arguably be the most difficult test she will ever experience—childbirth.

## Supplementary Information

Below is the link to the electronic supplementary material.

Supplementary file1 (DOC 170 KB)

## Data Availability

All data used for this review are published in the main manuscript and supplementary material.
