# Supplementary material for: Experiences of Health Facility Childbirth in Sub-Saharan Africa: A Systematic Review of Qualitative Evidence
Source: Matern Child Health J. 2022 Feb 26;26(3):481–92. doi: 10.1007/s10995-022-03383-9 (PMC8917011; doi:10.1007/s10995-022-03383-9)
Supplement: Supplementary file 1 — Supplementary file1 (DOC 170 KB) [file 10995_2022_3383_MOESM1_ESM.doc]

**Table S1**. PRISMA Checklist

| **Section/topic** | | **#** | | **Checklist item** | **Reported on page #** |
| --- | --- | --- | --- | --- | --- |
| **TITLE** | | | | |  |
| Title | | 1 | | Identify the report as a literature review. | 1 |
| **ABSTRACT** | | | | |  |
| Structured summary | | 2 | | Provide a structured summary including, as applicable: background; objectives; data sources; study eligibility criteria, participants, and interventions; study appraisal and synthesis methods; results; limitations; conclusions and implications of key findings; | 1 |
| **INTRODUCTION** | | | | |  |
| Rationale | | 3 | | Describe the rationale for the review in the context of what is already known about your topic. | 1-2 |
| Objectives | | 4 | | Provide an explicit statement of questions being addressed with reference to participants, interventions, comparisons, outcomes, and study design (PICOS). | 2 |
| **METHODS** | | | | |  |
| Eligibility criteria | | 5 | | Specify study characteristics (e.g., PICOS, length of follow-up) and report characteristics (e.g., years considered, language, publication status) used as criteria for eligibility, giving rationale. | 2 |
| Information sources | | 6 | | Describe all information sources (e.g., databases with dates of coverage) in the search and date last searched. | 2 |
| Search | | 7 | | Present full electronic search strategy for at least one database, including any limits used, such that it could be repeated. | 2 |
| Study selection | | 8 | | State the process for selecting studies (i.e., screening, eligibility). | 2 |
| Risk of bias in individual studies | | 9 | | Describe methods used for assessing risk of bias of individual studies (including specification of whether this was done at the study or outcome level). | N/A |
| Risk of bias across studies | | 10 | | Specify any assessment of risk of bias that may affect the cumulative evidence (e.g., publication bias, selective reporting within studies). | N/A |
| **RESULTS** | | | | |  |
| Study selection | 11 | | Give numbers of studies screened, assessed for eligibility, and included in the review, with reasons for exclusions at each stage, ideally with a flow diagram. | | 3-4 |
| Study characteristics | 12 | | For each study, present characteristics for which data were extracted (e.g., study size, PICOS, follow-up period) and provide the citations. | | 3-4 |
| Synthesis of results of individual studies | 13 | | For all outcomes considered (benefits or harms), present, for each study: (a) summary of results and (b) relationship to other studies under review (e.g. agreements or disagreements in methods, sampling, data collection or findings). | | 5 |
| **DISCUSSION** | | | | |  |
| Summary of evidence | 14 | | Summarize the main findings including the strength of evidence for each main outcome; consider their relevance to key groups (e.g., healthcare providers, users, and policy makers). | | 6 |
| Limitations | 15 | | Discuss limitations at study and outcome level (e.g., risk of bias), and at review-level (e.g., incomplete retrieval of identified research, reporting bias). | | 6 |
| **CONCLUSION** | | | | |  |
| Conclusions | 16 | | Provide a general interpretation of the results in the context of other evidence, and implications for future research. | | 7 |

*Adapted from:* Moher D, Liberati A, Tetzlaff J, Altman DG, The PRISMA Group (2009). Preferred Reporting Items for Systematic Reviews and Meta-Analyses: The PRISMA statement*. PLoS Medicine*, 6(6), e1000097.

**Table S2**. Full search strategy applied for peer review literature

10 Dec 2019

| Database | Key word combination |
| --- | --- |
| Embase  PubMed  Scopus | **#1** “mother*” OR “women” OR “matern*”  **#2** “health facility childbirth*” OR “health facility deliver*” OR “facility based childbirth*” OR “facility based deliver*” OR “hospital based childbirth*” OR “facility birth” OR “hospital based deliver*” OR “hospital birth” OR “institutional birth*” OR “institutional childbirth*” OR “institutional deliver*”  **#3** “childbirth experience” OR “birth experience” OR “experience of labour” OR “delivery experience”  **#4** “perception of birth” OR “perspective*” OR “views” OR “feel*” OR “understand*”  **#5** sub-Saharan Africa OR Angola OR Benin OR Botswana OR Burkina Faso OR Burundi OR Cameroon OR “Cape Verde” OR “Central African Republic” OR Chad OR Comoros OR Congo OR “Democratic Republic of Congo” OR” “Côte d'Ivoire” OR Djibouti OR “Equatorial Guinea” OR Eritrea OR Ethiopia OR Gabon OR “The Gambia” OR Ghana OR Guinea OR “Guinea-Bissau” OR Kenya OR Lesotho OR Liberia OR Madagascar OR Malawi OR Mali OR Mauritania OR Mauritius OR Mozambique OR Namibia OR Niger OR Nigeria OR Réunion OR Rwanda OR “Sao Tome and Principe” OR Senegal OR Seychelles OR “Sierra Leone” OR Somalia OR “South Africa” OR Sudan OR Swaziland OR Tanzania OR Togo OR Uganda OR “Western Sahara” OR Zambia OR Zimbabwe  #1 AND #2 AND #3 AND #4 AND #5 |

**Table S3**. Quality assessment of included studies

| **S/N** | **Authors and date** | **Was there a clear statement of the aims of the research?** | **Is a qualitative methodology appropriate?** | **Was the research design appropriate to address the aims of the research?** | **Was the recruitment strategy appropriate to the aims of the research?** | **Was the data collected in a way that addressed the research issue?** | **Has the relationship between researcher and participants been adequately considered?** | **Have ethical issues been taken into consideration?** | **Was the data analysis sufficiently rigorous?** | **Is there a clear statement of findings?** | **How valuable is the research?** | **CASP score (n/10)** |
| --- | --- | --- | --- | --- | --- | --- | --- | --- | --- | --- | --- | --- |
| 1 | Adinew and Assefa, 2017 | 1 | 1 | 1 | 1 | 1 | 0 | 1 | 1 | 1 | 1 | **9** |
| 2 | Afulani, Kurmbi and Lyndon, 2017 | 1 | 1 | 1 | 1 | 1 | 1 | 1 | 0 | 0 | 1 | **8** |
| 3 | Balde et al., 2017 | 1 | 1 | 1 | 1 | 1 | 0 | 1 | 1 | 1 | 1 | 9 |
| 4 | Bohren et al., 2016 | 1 | 1 | 1 | 1 | 1 | 1 | 1 | 1 | 1 | 1 | **10** |
| 5 | Bohren et al., 2017 | 1 | 1 | 1 | 1 | 1 | 0 | 0 | 1 | 1 | 1 | **8** |
| 6 | D'ambruso, Abbey & Hussein, 2005 | 1 | 1 | 1 | 0 | 1 | 1 | 1 | 0 | 0 | 0 | **6** |
| 7 | Dzomeku, van Wyk, and Lori, 2017 | 1 | 1 | 1 | 1 | 1 | 0 | 1 | 1 | 0 | 1 | **8** |
| 8 | Kumbani et al., 2012 | 1 | 1 | 1 | 0 | 1 | 1 | 1 | 1 | 1 | 1 | **9** |
| 9 | Madula et al., 2018 | 1 | 1 | 1 | 1 | 0 | 0 | 1 | 0 | 0 | 1 | 6 |
| 10 | Maputle and Nolte, 2008 | 1 | 1 | 1 | 0 | 0 | 0 | 1 | 0 | 0 | 1 | **5** |
| 11 | Maya et al., 2018 | 1 | 1 | 1 | 0 | 1 | 1 | 1 | 1 | 1 | 1 | 9 |
| 12 | McMahon et al., 2014 | 1 | 1 | 1 | 1 | 1 | 0 | 1 | 1 | 1 | 1 | **9** |
| 13 | Mensah, Mogale and Ritcher, 2014 | 1 | 1 | 1 | 0 | 1 | 0 | 1 | 0 | 0 | 0 | **5** |
| 14 | Mukamurigo et al, 2017 | 1 | 1 | 1 | 1 | 1 | 0 | 1 | 1 | 0 | 1 | **8** |
| 15 | Muntenda, Nuuyoma and Stern, 2017 | 1 | 1 | 1 | 1 | 0 | 0 | 1 | 0 | 0 | 1 | **7** |
| 16 | Namujju et al, 2018 | 1 | 1 | 1 | 1 | 1 | 0 | 1 | 0 | 1 | 1 | **8** |
| 17 | Nwosu et al, 2012 | 0 | 1 | 1 | 0 | 0 | 0 | 1 | 0 | 0 | 1 | **4** |
| 18 | Ojelade et al, 2017 | 1 | 1 | 1 | 1 | 1 | 0 | 1 | 1 | 1 | 1 | **9** |
| **19** | Okonofua et al 2017 | 1 | 1 | 1 | 0 | 1 | 0 | 0 | 0 | 1 | 1 | **6** |
| 20 | Okwako and Symon, 2014 | 1 | 1 | 1 | 1 | 1 | 0 | 0 | 0 | 1 | 1 | **7** |
| **21** | Orpin et al, 2018 | 1 | 1 | 1 | 1 | 1 | 0 | 1 | 0 | 1 | 1 | **8** |
| **22** | **S**himpuku et al, 2013 | 1 | 1 | 1 | 0 | 1 | 0 | 1 | 0 | 0 | 1 | **6** |

;

| **Low** | **Average** | **High** |
| --- | --- | --- |

| **Table S4: Supporting quotes from included studies** | |
| --- | --- |
| **Experience of Care Domain** | **Supporting quotes** |
| **4. Effective communication** | |
| 4.1 All women and their families receive information about the care and have effective interactions with staff | *They were also next to me and told me that I will give birth at six in the morning and they were awake the whole night and asked me to call them anytime …they made me happy and this even reduced the amount of pain that I was going through because they kept on encouraging me and this made me feel welcomed”* **(Woman, Kenya, Afulani, Kurumbi and Lyndon, 2017)**  *“All they said was we are done after examining you. The results they keep to themselves after examining you. You are just told to go when they are done.”* **(Woman, Malawi, Kumbani et al.,2012)**  *"I was not involved and not informed about my progress, the midwives will examine me, and I will only overhear them giving each other the report, but not telling me so that I can participate.” “They know what to do and how to care for us during labour but at times I just felt I’m not involved because I’m just told what to do without being asked. I don’t want to be restricted”*. **(Woman, South Africa, Maputule and Nolte, 2008)**  *"I was feeling like a cow they were taking to slaughter, when people are looking at things without explaining to you and you don’t see anything. When a person doesn’t tell you that you have a certain problem, you wonder if you will die or live. You think that there is something that they are hiding from you".* **(Woman, Rwanda, Mukamurigo et al., 2017)**  "*You know I don’t understand Yoruba that much, so I just have to tell them, see I don’t understand what you are saying, you understand, thank God the person attending to me just start breaking it down, break everything down for my own understanding, you get it?* " **(Woman, Nigeria, Ojelade et al., 2017)**    *"Communication is very vital…I think that’s just what we are lacking in this government facility…the communication channel there is broken already. We need to try and bring it back to normal the way it is supposed to be. Let’s teach them how to communicate with people as if they are the owner of this health facility, because if you are the owner of something you want to make it good"* **(Woman, Nigeria, Ojelade et al., 2017)**  *"All I need is just the truth and everything. Sometimes when they write something with their big grammar, they feel you don’t understand, when you ask them, they will say ‘don’t worry.’ I don’t feel good about that. I think they feel everybody doesn’t know anything. No matter how big the grammar is, try and explain to the person what it is or what the problem is".* **(Woman, Nigeria, Bohren et al., 2017)** |
| 4.2 All women and their families experience coordinated care, with clear, accurate information exchange between relevant health and social care professionals. | *“[The best thing was] the good welcome…. I arrived in the hospital at 1.00 am at night, the watchman opened the gate well, he welcomed me and called the nurse, then she welcomed me also…when I arrived, I found everything ready. She took me to the bed, did everything, told me now it is late the chemist is closed, we do not have any drug here, but she looked for some of her personal drugs and used them on me... She took her time to be with me and handled me well. This made me to say it is good to go to the hospital to deliver.”* **(Woman, Kenya, Afulani, Kurumbi and Lyndon, 2017)**    *“They did not tell me anything, I just saw the vehicle ready and they told me you are going to [X referral hospital] because you are going for an operation.”*  **(Woman, Kenya, Afulani, Kurumbi and Lyndon, 2017)**     *"They [healthcare providers] should continuously ask questions… ‘how do you feel,’ ‘how are you feeling now.’ It’s not supposed to just be the woman that will be telling them ‘please come check on me’…they [healthcare providers] should be continuously telling the woman ‘this is your condition,’ and educate them"* **(Woman, Nigeria, Ojelade et al., 2017)**     *"What I know is that they don’t have good manner of approach, they always like to harass persons here. If you ask questions, it is just like a big sin. When you are asking questions, you need to know your left and right. I don’t know the way they are acting here. I don’t understand! Even since yesternight I was here telling them to come and do the test, they didn’t come, they were telling me they will come, they will come, but the pain is increasing. Because of the pain, I did not sleep well yesterday even to this afternoon the same thing, for me to eat now is a problem, I only take something liquid, how will I get well*!" **(Woman, Nigeria, Okonofua et al., 2017)** |
| **5. Respect and preservation of dignity** | |
| 5.1 All women and newborns have privacy around the time of labour and childbirth, and their confidentiality is respected | *“The curtains were drawn so that people passing by would not see what was happening inside”* ***(Woman, Malawi, Kumbani et al.,2012)***  *“In the hospital … they are making you lie on the bed to be examined, there is no sheet to cover you and … other people are passing there. They will uncover you and sometimes you remain with a petty coat or sometimes you do not have one, you remain with a panty and they have not covered you, this sometimes embarrasses me...”*  **(Woman, Kenya, Afulani, Kurumbi and Lyndon, 2017)**    *“In health center there is no privacy at all. Everybody who comes in and out of the delivery room watches my naked body and inserts their fingers into my genitalia including the male. Had it been at home let alone inserting finger no one will see my privates as the areawill be dark enough to provide me the maximum privacy and only females will attend me*” **(Woman, Ethiopia, Adinew and Assefa, 2017)**  *“I want a private area, there was a time…what drove me away from using that hospital is the delivery facility, the delivery room is like a big hall where about eight or six delivery couches are there and they were not screened. I was like, you are having your own issues, the other person is having his or her own, everybody. So I prefer where they have one delivery couch, just be there with the doctor and the Nurse”* **(Woman, Nigeria, Bohren et al., 2017)**    “*Where we give birth in this general hospital is not good because up to four people can go through labor in a single room…. nurses and visitors will also be in the same room… sleeping on the floor, this is not adequate.”* **(Woman, Nigeria, Ojelade et al., 2017)**  *"now you are under stress and you feel like… they have to do it (operation) immediately. “They take you in the theatre room, you again spend some good time there when they are still organizing. It was too bad, ….now even you feel stress is coming back again , you try to console yourself , you control…As you wait, you are naked, you are exposed there… (laughed while shaking her head)… you know how funny it is... you are nude. It was not good; privacy was not enough”*. **(Woman, Uganda, Namujju et al., 2018)** |
| 5.2 No woman or newborn is subjected to mistreatment, such as physical, sexual or verbal abuse, discrimination, neglect, detainment, extortion or denial of services. | *“When my time for delivery reached, it was at around eleven in the night. So, when I came…I tried to knock the gate and even shout at the watchman to come and open for me, but he did not. So, I gave birth right at the gate… the doctor came at half past midnight and… the baby’s umbilical cord was cut outside at the hospital gate…*.” **(Woman, Kenya, Afulani, Kurumbi and Lyndon, 2017).**  *"The nurse gets angry. She tells you, ‘You have already delivered many children. This is enough! Look at the others who have delivered only twice or thrice and stopped!’ You will (be in the middle of labor) and hear the nurse saying, ‘Come and stop having children!"* **(Woman, Tanzania, McMahon et al.,2014)**  *"There is no place you can go; you must keep quiet. …They can hurt you… The routine is set. …. I’m afraid that if I say anything to anyone, I could get reported or not get treatments."* **(Woman, Tanzania, McMahon et al.,2014)**  *Some of service providers are hostile to a labouring mother. For instance, when I gave birth to my first child in the health center, I was ashamed to open my leg in front of male nurses during labour. Then one of the health workers said, “had it been for sex you would open shamelessly.” It was my fault, had it been in my mom’s home I would enjoy the ceremony and give birth with respect. But I refused them and went to health center just to end up with such insult. This type of arrogant approaches made me to give birth to my second child at home.* **(Woman, Ethiopia, Adinew and Assefa, 2017)**  *"The traditional birth attendants will take care of your feelings; they treat you with sympathy. They are well aware of and concerned about our culture, so they never do something that can disappoint you. But those in health facilities act as if they were from another planet. They enjoy your pain and degrade you from humanity. I don’t even understand why they are here if they don’t respect and serve the needy".* **(Woman, Ethiopia, Adinew and Assefa, 2017)**  *It is true! Women are slapped by nurses in the ward. For me they did not, but I witnessed a mother who delivered in the toilet. She was told to go and shower but did not make it, and the baby dropped! The nurse ran to her and slapped her, because she was putting the nurses into trouble".* **(Woman, Namibia, Muntenda, Nuuyoma and Stern, 2017)**  *“Sometimes there is even no reason for the abusive language, but it is a common thing at the hospital”* **(Woman, Nigeria, Orpin et al., 2018)**  *“When they pinch them it means they don’t obey the instructions of the nurses. Maybe the baby is coming, and you are closing your legs so the nurse must pinch you to open them up. Yes, it is acceptable”* **(Woman, Ghana, Maya et al., 2018)**  *“ Sometimes is not the fault of the health worker. When it is time for you to push the baby out and you don’t push, that one it is not the fault of the health worker. If she doesn’t slap, you to push the baby out you will not [push]. Sometimes it is our fault”* **(Woman, Ghana, Maya et al., 2018)**  *Some [women] are difficult, refuse to obey doctor’s recommendations, not to harm the baby, the doctor can get upset against the woman because he is scared of the baby’s death***(Woman, Guinea, Balde et al., 2017)**  *There are difficult women, some of us stand, bend, kneel or hop. They hit you, or bend on you, they tell you, “you have to lie down here to avoid issues to the baby.” At that time, if they insult me or hit it is acceptable because it is to help me.* **(Woman, Guinea, Balde et al., 2017)** |
| 5.3 All women have informed choices in the services they receive, and the reasons for interventions or outcomes are clearly explained. | “I was given medicine… I do not know whether it was Bactrim or Panadol... They just asked who delivered today and gave us the medicine. I do not know what it was for. I just received since I am in hospital”. ***(Woman, Malawi, Kumbani et al.,2012)*** ‘‘One nurse told me that they wanted to insert a [vaginal pessary] which sometimes causes problems. But the doctor decided to allow me to go through labor naturally. She [the nurse] promised to be with me so I should not be [afraid]. I felt good and really happy because I had someone to depend on.’’ ***(Woman, Ghana, Mensah, Mogale and Ritcher, 2014)*** "The doctor told me, Irene, with you, you are just going for an operation. …I had to break down…because I was like what has gone wrong? ... why not me to deliver like other women? ….they are telling me I am going for an operation but they are not telling me the cause! Doctor Just told me, the baby was big. He then used some medical language that I did not understand. She did not convince me as to why I should have an operation… they were talking alone! **(Woman, Uganda, Namujju et al., 2018)**    "There was a time that my baby did not go past 6 centimeters [i.e. the woman’s cervix was not dilating], so I heard them discussing some kind of things… so I called the nurse that was attending to me… that does that mean they have to tear me and all that, so they said no, no, no, so she told me one thing that see, what we are doing here, what we are saying here does not concern you, we might just be talking, that is our own, but for, for me here, say before they do anything they should seek my consent, they cannot just come and do whatever they want, before they do anything they should seek my consent and that of my family that came with me" ***(Woman, Nigeria, Ojelade et al., 2017)*** |
| **6. Emotional support** | |
| 6.1 Every woman is offered the option to experience labour and childbirth with the companion of her choice. | *"They should let us give birth in presence of our husbands. This strengthens our security"* **(Woman, Rwanda, Mukamurigo et al.,2017)**   *“When I came back to the labour ward, they told me “the baby has reached you push”, I was not feeling any energy. ….my husband helped me, held me and he never feared. When the baby was coming out, he told me that “bambi” (meaning my friend) push more, the head is coming, add in more effort. … I felt good, I liked it so much because he gave me support, and he was there*” **(Woman, Uganda, Namujju et al., 2018).**    *“During antenatal classes, they told us that they allow our husbands in the labor ward, but they stopped my husband at the reception and they never allowed him in. Yet I needed to hold him for comfort, but they were like ‘if you need to hold some thing, just hold the bed.’ Now, I didn’t find any comfort in holding the bed. She [midwife] could not provide a hand and she just chased my husband out of the labor ward.”* **(Woman, Uganda, Bohren et al., 2017)**   *I think women should be allowed their husbands in…my husband was right there with me; my first delivery, it was painful but with his encouragement, he was there holding my hands, doing this, even when the doctors were telling madam push, push, I didn’t listen to the doctors but when my husband say madam push, push, that is when I started pushing. I think it is a psychological thing when your husband is right there with you* **(Woman, Uganda, Bohren et al., 2017).** |
| 6.2 Every woman receives support to strengthens her capability during childbirth. | *"The services were not so good, the attendant ... refused when I needed to hold her while I was in pain she said it won't change anything...even when I asked the ward assistant for water she brought me chilled water, when I said I preferred tap water, she became angry."* (**Woman, Ghana, d'Ambruso, Abbey and Hussein, 2005)**   *"The nurse doesn’t allow anyone to enter inside the room. She is usually alone or maybe with another nurse. I never saw any help (during delivery). You must prepare yourself and just go [Laughing] … you can’t blame anyone. That nurse’s condition is hard".* **(Woman, Tanzania, Mcmahon et al., 2014)**   *‘‘Everybody [implying the nurses in that unit] who came around encouraged me. . .some massaged my back and also [showed] patience with me. Oh God! these nurses!! I had the right people to support me.’*’ **(Woman, Ghana, Mensah, Mogale and Ritcher, 2014)**.   *"Good quality of care is when you come to a health facility, you are received, they know what that moment means to you and that of your child and the kind of reception they give you as a mother that wants to deliver her child. They receive you warmly, encourage you, if you are having pain anywhere you know that they will help you, not that the pains will go away but the words they give and what they do to you helps you to have that courage. You are in good hands, what matters most is that when you come into a health facility, there’s this confidence derived that assures one that she’s in good hands".* **(Woman, Nigeria, Bohren et al., 2017)**  “The nurses were not enough…Also even though the nurses were not enough they sat by their table too much. If they could move around the labour ward, they will know who needs help. One woman delivered on the bed alone in the labour room because we are not well monitored” **(Woman, Ghana, Maya et al., 2018)** |

**Table S5: Further characteristics of included studies**

| S/N | **Authors** | **Date** | **Country** | **Aims/Objectives of the study** | **Place of recruitment** | **Interval from birth to interview** |
| --- | --- | --- | --- | --- | --- | --- |
| 1 | Adinew, and Assefa | 2017 | Ethiopia | To explore why some women who had previous experience of facility based delivery care gave birth at home for their most recent child by in-depth understanding of women’s previous facility based delivery experience, perspective towards health facilities and service providers with regard to delivery services. | Communities | 12 months or less |
| 2 | Afulani, Kurmbi and Lyndon | 2017 | Kenya | To examine women’s facility–based childbirth experiences in a rural county in Kenya, to identify aspects of care that contribute to a positive or negative birth experience. | Communities | 9 weeks or less |
| 3 | Balde et al. | 2017 | Guinea | To explore women’s and health providers’ attitudes and acceptability of mistreatment during childbirth in health facilities in Guinea | Communities | 12 months or less (IDI) 5 years or less (FGD) |
| 4 | Bohren et al. | 2016 | Nigeria | To better understand the social norms and acceptability of the mistreatment of women during childbirth in Abuja, Nigeria | Communities | 12 months or less (IDI) 5 years or less (FGD) |
| 5 | Bohren et al. | 2017 | Nigeria and Uganda | To explore what “quality of care” means to Nigerian and Ugandan women, particularly focusing on the “experience of care” quality domains of the WHO framework. | Communities | 12 months or less (IDI) 5 years or less (FGD) |
| 6 | D'ambruso, Abbey & Hussein | 2005 | Ghana | To explore women's accounts of maternity services during labour and delivery in Ghana | Health facilities. | within 5 years |
| 7 | Dzomeku, van Wyk, and Lori | 2017 | Ghana | The aim of this study is to add to a growing body of literature on disrespect and abuse during facility based childbirth through an exploratory study on how mothers encountered childbirth care at four public health facilities in Ghana. | Health facilities | six weeks or less |
| 8 | Kumbani et al. | 2012 | Malawi | The objective of the study was to describe perceptions of perinatal care among women who delivered at a district hospital in Malawi | Health facility | 1 - 7 days |
| 9 | Madula et al. | 2018 | Malawi | The objective of this study was therefore, to explore the nature of communication between healthcare providers and pregnant women in some hospitals in Malawi with the view to establishing whether communication is one of the reasons behind women’s choice to deliver at a health facility or not | Health facilities | pregnant women and women in postnatal wards |
| 10 | Maputle and Nolte | 2008 | South Africa | The aim of this study was to explore and describe experiences of mothers during childbirth in a tertiary hospital in the Limpopo Province. | Health facility | Women in post natal wards |
| 11 | Maya et al. | 2018 | Ghana | To explore women’s perspectives of mistreatment during facility-based childbirth in the Ghanaian context specifically. | Communities | 12 months or less |
| 12 | McMahon et al. | 2014 | Tanzania | To explore how rural Tanzanian women and their male partners describe disrespect and abuse experienced during childbirth in facilities and how they respond to abuse in the short or long-term. | Communities | 14 months or less |
| 13 | Mensah, Mogale and Ritcher | 2014 | Ghana | What were the experiences of the birthing women at the 37th Military hospital when compared with their previous labor and delivery experiences at home or in other health care settings? | Health facility | 48 hours or more |
| 14 | Mukamurigo et al. | 2017 | Rwanda | To explore the meaning of a poor childbirth experience as expressed by women who had given birth in Rwanda. | Communities | 13 months or earlier |
| 15 | Muntenda, Nuuyoma and Stern | 2017 | Namibia | To explore the perceptions of women on child birthing in a public-health facility in a peri-urban area in Kavango east region, Namibia | Health facility | Post natal clinics (unspecified) |
| 16 | Namujju et al. | 2018 | Uganda | To explore the childbirth experiences and the perceived meanings among postnatal mothers seeking postnatal services at Mbale Regional Referral Hospital in Eastern Uganda. | Health facility | Two months or less |
| 17 | Nwosu et al. | 2012 | Nigeria | To determine the attitude of women towards private and public hospitals in accessing obstetric care in Nnewi, South-East Nigeria and evaluate its implications for maternal mortality reduction efforts in the country. | Communities | 12 months or less |
| 18 | Ojelade et al. | 2017 | Nigeria | To explore women's needs for communication and emotional support during facility based delivery | Communities | 12 months or less |
| 19 | Okonofua et al. | 2017 | Nigeria | To investigate women’s level of satisfaction with the quality of care in the three dimensions of structure, process and outcomes in the continuum of maternity care - antenatal, intrapartum, and postnatal services. The secondary aim was to identify, based on recommendations by the women, cost-effective measures that could be put in place to improve women-friendly approaches for the management of obstetric services in secondary and tertiary health institutions in Nigeria. | Health facilities | Antenatal or postnatal clinics (unspecified) |
| 20 | Okwako and Symon | 2014 | Kenya | To explore the childbirth expectations and experiences of a small group of multiparous women in a Kenyan public hospital | Health facility | 24 hours |
| 21 | Orpin et al. | 2018 | Nigeria | To explore women’s perceptions and experiences of Disrespect and abuse in maternity care facilities in Benue State, Nigeria. | Health facilities | two years but specific for maltreatment |
| 22 | **S**himpuku et al. | 2013 | Tanzania | To explore women’s perceptions about their hospital birth experience. Women were interviewed after giving birth at a hospital to identify the following: (a) what they experienced during attended births, (b) how they assessed this birthing experience, and (c) what attracted the women to deliver in the presence of SBAs. | Health facility | 24 hours |
